# A new phenotypic classification system for dyslipidemias based on the standard lipid panel

**DOI:** 10.1186/s12944-021-01585-8

**Published:** 2021-11-27

**Authors:** Maureen Sampson, Rami A. Ballout, Daniel Soffer, Anna Wolska, Sierra Wilson, Jeff Meeusen, Leslie J. Donato, Erica Fatica, James D. Otvos, Eliot A. Brinton, Robert S. Rosenson, Peter Wilson, Marcelo Amar, Robert Shamburek, Sotirios K. Karathanasis, Alan T. Remaley

**Affiliations:** 1 Department of Laboratory Medicine, Clinical Center, National Institutes of Health, Bethesda, MD USA; 2 Lipoprotein Metabolism Laboratory, Translational Vascular Medicine Branch, National Heart, Lung, and Blood Institute, National Institutes of Health, 10 Center Drive, Bldg. 10/Rm. 2C433, Bethesda, MD 20892 USA; 3 Department of Internal Medicine, Perelman School of Medicine at the University of Pennsylvania, Philadelphia, PA USA; 4 Department of Laboratory Medicine and Pathology, Mayo Clinic, Rochester, MN 55905 USA; 5 Laboratory Corporation of America Holdings (LabCorp), Burlington, NC USA; 6 Utah Lipid Center, Salt Lake City, UT USA; 7 Cardiometabolics Unit, Zena and Michael A. Wiener Cardiovascular Institute, Icahn School of Medicine at Mount Sinai, New York, NY USA; 8 Atlanta VAMC and Emory Clinical Cardiovascular Research Institute, Atlanta, GA 30322 USA

**Keywords:** Cholesterol, LDL, Lipids, Lipoproteins, Genetics, Cardiovascular disease

## Abstract

**Background:**

Dyslipoproteinemias can be classified by their distinct lipoprotein patterns, which helps determine atherosclerotic cardiovascular disease (ASCVD) risk and directs lipid management but this has required advanced laboratory testing.

**Objective:**

To develop a new algorithm for classifying lipoprotein disorders that only relies on the standard lipid panel.

**Methods:**

Lipid thresholds for defining the different lipoprotein phenotypes were derived for Non-High-Density Lipoprotein-Cholesterol (NonHDL-C) and Triglycerides (TG) to be concordant when possible with the current US Multi-Society guidelines for blood cholesterol management.

**Results:**

The new classification method categorizes patients into all the classical Fredrickson-like phenotypes except for Type III dysbetalipoproteinemia. In addition, a new hypolipidemic phenotype (Type VI) due to genetic mutations in apoB-metabolism is described. The validity of the new algorithm was confirmed by lipid analysis by NMR (*N* = 11,365) and by concordance with classification by agarose gel electrophoresis/beta-quantification (*N* = 5504). Furthermore, based on the Atherosclerosis Risk in Communities (ARIC) cohort (*N* = 14,742), the lipoprotein phenotypes differ in their association with ASCVD (TypeV>IIb > IVb > IIa > IVa > normolipidemic) and can be used prognostically as risk enhancer conditions in the management of patients.

**Conclusions:**

We describe a clinically useful lipoprotein phenotyping system that is only dependent upon the standard lipid panel. It, therefore, can be easily implemented for increasing compliance with current guidelines and for improving the care of patients at risk for ASCVD.

**Supplementary Information:**

The online version contains supplementary material available at 10.1186/s12944-021-01585-8.

## Highlights

A new algorithm is described for categorizing dyslipidemic patients into Fredrickson-like lipoprotein phenotypes except for Type III.

The new lipoprotein phenotypes were validated by NMR-lipoprotein analysis and by agarose gel electrophoresis/beta-quantification in a large number of subjects.

The new lipoprotein phenotyping system identifies high-risk cardiovascular patients and helps direct clinical management.

A major advance is that the new lipoprotein phenotypes are based on just the standard lipid panel, and thus can be automatically calculated by the clinical laboratory and widely implemented.

**Supplementary Information:**

The online version contains supplementary material available at 10.1186/s12944-021-01585-8.

## Background

Elevated plasma lipids, in particular total cholesterol (TC) and triglycerides (TG), increase the risk of atherosclerotic cardiovascular disease (ASCVD); hence, their measurement is integral to ASCVD risk assessment and prevention [1]. The levels of TC and TG in plasma are influenced by a complex network of metabolic pathways, which when disturbed by disease or environmental influences, will alter the concentration of the various lipoproteins that transport these lipids in the circulation. Perturbations in the normal physiologic level of plasma lipoproteins were first noted over 50 years ago by Donald S. Fredrickson and colleagues, whose observations became the foundation for the first phenotypic classification of lipoprotein disorders [2–6].

The three main classes of apolipoprotein B (apoB)-containing lipoprotein particles are Low-Density Lipoproteins (LDL), Very-Low Density Lipoproteins (VLDL) and chylomicrons. Nearly all possible permutations for elevations in these lipoproteins, taken one or two at a time, comprise the classic Fredrickson classification system. Three of these phenotypes are characterized by an increase in a single type of lipoprotein, namely Type I (chylomicrons), Type IIa (LDL), and Type IV (VLDL). In the Types IIb and V phenotypes, two classes of lipoproteins are increased, and thus they are sometimes called mixed dyslipoproteinemias. In Type IIb, there is an increase in both VLDL and LDL, whereas in Type V both VLDL and chylomicrons are increased. The only other possible permutation for simultaneous elevations in two lipoprotein classes would be an increase in LDL and chylomicrons, but this pattern has only been described in a single case report [7]. The other remaining Fredrickson phenotype is Type III. It is a relatively uncommon disorder characterized by the accumulation of cholesterol-enriched remnant particles, due to impaired apoE-mediated hepatic clearance of partially lipolyzed VLDL and chylomicrons [8]. It is important to note that the Fredrickson classification system does not address dyslipidemias related to low HDL-C or elevated Lp(a).

The Fredrickson classification was originally established by separating lipoproteins by density gradient ultracentrifugation (beta-quantification), but later it was mostly performed by the more convenient method of agarose gel electrophoresis [9]. Although the classification of Fredrickson lipoprotein phenotypes is still used for didactic purposes, it is currently only available in specialty reference laboratories and is no longer widely used in clinical practice [9]. The gradual discontinuation of detailed lipoprotein phenotyping, starting in the late 1980s, was largely driven by the advent of statin therapy. Contemporary guidelines have almost exclusively focused on reducing LDL-C with statins as the initial approach for ASCVD risk reduction by lipid-lowering therapy.

In 2007, Allan D. Sniderman and colleagues described a modification of the Fredrickson classification system in which apoB, along with TC and TG, were used as surrogate measures of lipoprotein concentrations [10]. They also made several well-reasoned recommendations on how lipoprotein phenotyping could enhance the clinical management of patients in the modern era, including the use of various lipid therapies that alter other lipid parameters besides LDL-C [11]. Since that time, additional lipid-lowering drugs have been approved, particularly for hypertriglyceridemia, which may benefit from a more refined classification of lipoprotein phenotypes [12, 13].

The current 2018-Multiscociety Blood Cholesterol Guideline [1] and the National Lipid Association (NLA) Recommendations [14] on lipid management for the primary prevention of ASCVD, still mostly depend on the standard lipid panel (TC, TG, and HDL-C), which can be used to estimate LDL-C [15]. Although ApoB can be accurately measured and can improve ASCVD risk assessment [16, 17], it is not recommended as a primary screening test by current US guidelines. This decision was made, in part, in order to simplify the screening process and because the addition of apoB to the standard lipid panel testing would approximately double the cost for ASCVD-risk screening [18].

In an effort both to be cost-effective and to conform with existing US guidelines, we have developed a new algorithm for lipoprotein phenotyping based on just two components of the standard lipid panel, namely TG and NonHDL-C (TC minus HDL-C). The new classification system depends upon 7 lipid cut-points already recommended by the 2018-Multiscociety Guideline for patient management [1], plus two non-traditional lipid-based rules. Although this new classification system cannot be used for identifying Type III dyslipidemia, it does identify the other more common Fredrickson phenotypes related to apoB-containing lipoproteins. A new phenotype, Type VI, which occurs due to rare genetic disorders in apoB-metabolism and requires a specific treatment, is also described. Finally, we show how this new lipoprotein phenotyping system can serve as a general guide or framework, particularly for the non-lipid specialist, in the diagnosis and clinical management of patients at risk for ASCVD.

## Materials and methods

### Study design and biochemical measurements

Laboratory, observational study lipid databases, and ASCVD outcome experience from large observational cohorts were used to develop, cross-validate, and examine the potential utility of the new lipoprotein phenotyping method. Research under this study was considered non-human subject research and was exempted by the NIH-IRB.

Standard lipid panel tests (*N* = 32,696), measured by standard enzymatic methods, plus apoB, were performed on the Cobas 6000 analyzer (Roche) on patients (*N* = 14,120) tested on multiple occasions at the NIH Clinical Center from 2013 to 2018. Patients in the study included those with a normal lipoprotein profile and also a wide variety of rare lipid disorders. Patients on lipid-lowering therapy were not excluded from analysis. NMR LipoProfile analysis (LabCorp) was also performed on a convenience-derived subset of the same samples (*N* = 11,365), using the Vantera NMR analyzer and LP4 deconvolution algorithms [19]. De-identified clinical laboratory test results from the National Health and Nutrition Examination Survey (NHANES, 2005–2016) (*N* = 13,086) were used for external validation and downloaded from the following website: https://wwwn.cdc.gov/nchs/nhanes/. De-identified datasets for external validation were also obtained from the Multi Ethnic Study of Atherosclerosis (MESA) (*N* = 6788) and the Atherosclerosis Risk in Communities (ARIC) (*N* = 14,742), using BioLINCC (https://biolincc.nhlbi.nih.gov/home/). Metabolic Syndrome was defined using 2004-AHA guidelines [20].

Routine diagnostic testing for lipoprotein phenotyping of patients (*N* = 5602) was performed at Mayo Clinic Laboratories, using a combination of agarose gel electrophoresis and beta-quantification (gel-lipid method). The lipid phenotype was assigned after reviewing agarose gel electrophoresis in the context of LDL-C as determined by beta-quantification (LDL-C_ßQ_) and other lipid values as described below. Serum was ultracentrifuged for 15 h at 75,000 x g to generate a supernatant with density < 1.006 g/L. The upper layer (containing chylomicrons and VLDL) and lower layer (containing HDL and LDL) were separated and transferred to aliquot tubes. TC and TG concentrations were determined enzymatically in whole serum and in both density layers. HDL-C was determined following selective precipitation of LDL from the lower layer, using a mixture of magnesium chloride and dextran sulfate. LDL-C_ßQ_ is calculated as ([lower layer cholesterol] – [HDL-C]). Phenotypes were assigned according to the following criteria: (1) Type I = abundant chylomicrons with minimal VLDL detected on gel and VLDL-layer Cholesterol/TG ratio (V_ratio_) < 0.1. (2) Type IIa = LDL-C_ßQ_ > 190 mg/dL and VLDL-layer TG < 120 mg/dL (mild IIa if LDL-C_ßQ_ = 160–189). (3) Type IIb = LDL-C_ßQ_ > 190 mg/dL and VLDL-layer TG > 120 mg/dL (mild IIb if LDL-C_ßQ_ = 160–189 mg/dL). (4) Type III = floating beta-VLDL on gel and VLDL-layer TG > 120 mg/dL and V_ratio_ > 0.33. (5) Type IV = LDL-C_ßQ_ < 160 mg/dL and VLDL-layer TG > 250 mg/dL (mild if VLDL-layer TG = 120–249 mg/dL). (6) Type V = chylomicrons and VLDL detected on gel and total plasma TG > 1000 mg/dL and V_ratio_ < 0.2.

### Statistical methods

Mean differences between groups were analyzed by ANOVA, or t-test, in case of two groups, with *P* < 0.05 considered statistically significant. Statistical analysis was performed using JMP software (SAS, NC). Software developed for lipoprotein phenotyping analysis by the newly developed method can be freely downloaded at the following website: https://figshare.com/articles/software/Sampson_Phenotype_Calculator/16617490

## Results

### Lipid cut-points for new classification system for lipoprotein phenotypes

**Fig. 1:**
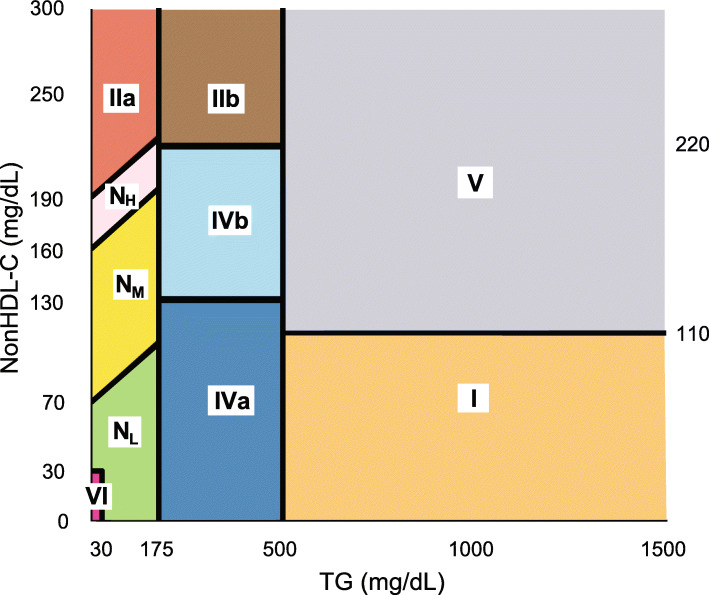
Diagram of new lipoprotein phenotyping classification system. Solid lines indicate various nonHDL-C and TG cut-points used to define various lipoprotein phenotypes (Type I-VI dyslipidemias, and the Normolipidemic subtypes (High (H), Moderate (M) and Low (L)). Phenotypes are color coded as indicated

A diagram of our new classification system for lipoprotein phenotypes is shown in Fig. 1 or as a flowchart in Supplemental Fig. 1. The two vertical TG cut-points of 175 and 500 mg/dL are based on the 2018-Multiscociety Guideline for lipids [1]. In these guidelines, TG > 175 mg/dL is used to define hypertriglyceridemia, which we also use to identify the 4 traditional hypertriglyceridemic phenotypes (Type I, IIb, IV, and V). When TG > 500 mg/dL, one should consider the use of TG- lowering therapy for reducing the risk of pancreatitis [1] and is the cut-point that the FDA uses for the approval of TG-lowering drugs for the prevention of pancreatitis. We used this same TG cut-point to distinguish Type I and V phenotypes, which are at increased risk of pancreatitis [21] [22], from Type IIb and Type IV, which have more moderate increases in TG and a lower risk for pancreatitis.

Most of the nonHDL-C cut-points are based on the following guidelines [1] for stratifying ASCVD risk by LDL-C: LDL-C ≥ 190 mg/dL, a criteria often used to define high risk Type IIa patients [23], LDL-C between 160 and 190 mg/dL (Normolipidemic High risk (N_H_)), LDL-C between 70 and 160 mg/dL (Normolipidemic Moderate risk (N_M_), and LDL < 70 mg/dL (Normolipidemic Low risk (N_L_). To convert these LDL-C limits into nonHDL-C units, the Friedewald equation was rearranged to calculate NonHDL-C (NonHDL-C = LDL-C + 0.2TG) and LDL-C was fixed at either 190, 160 or 70 mg/dL, thereby creating the three diagonal nonHDL-C boundary lines seen in Fig. 1.

For TG between 175 and 500 mg/dL, there are two additional horizontal nonHDL-C boundary lines (Fig. 1). The top line defines the boundary between Type IIb and Type IV and was fixed at a NonHDL-C of 220 mg/dL, the decision point recommended instead of LDL-C to initiate statin treatment in hypertriglyceridemic patients, according to 2015-NLA recommendations [14], which is also consistent with the current 2018-Multiscociety Guideline [1], as well as older US guidelines [24]. The lower NonHDL-C cut-point of 130 mg/dL is currently used as an alternative for LDL-C in defining patients at lower risk for ASCVD [1]. We used this cut-point for dividing the Type IV phenotype into two subtypes (Type IVa and Type IVb). As will be shown later, compared to Type IVb, subjects with Type IVa have a higher proportion of large Triglyceride-rich lipoprotein (TRL) particles than small TRL particles and are at a lower risk for ASCVD. We also use this cut-point because now that the 2018-Multisociety Guideline [1] has endorsed the use of non-fasting samples for screening, it is likely that many non-fasting patients may have plasma lipids that transiently fall into Type IVb phenotype but may have a more normolipidemic phenotype when fasting.

For TG ≥ 500 mg/dL, we have one horizontal boundary line at a fixed NonHDL-C value of 110 mg/dL (Fig. 1), a non-traditional lipid cut-point. We chose it, because it approximately corresponds to the apoB decision limit of 75 mg/dL (Supplemental Fig. 2), which is one of the rules used in the Sniderman classification [10] to distinguish Type I from Type V. We, therefore, define Type V as all patients with a TG ≥ 500 mg/dL and a NonHDL-C ≥ 110 mg/dL. This phenotype has the widest range of lipid values in our classification system, but it is sparsely populated. Unlike Type I, patients with this condition usually present in adulthood and are characterized as having high interindividual variability in their plasma lipids [25, 26], which can fall anywhere in the upper right quadrant of the plot (Fig. 1).

We also describe a new lipoprotein phenotype designated as Type VI. It is based on a compound lipid rule (NonHDL-C ≤ 30 mg/dL (≤0.02 ^st^ percentile) and a TG ≤ 30 mg/dL (≤1st percentile)) that is not used by any current guidelines (Fig. 1). We designed this new phenotype to identify hypolipidemic patients not on lipid-lowering treatment, who have genetic disorders in apoB-containing lipoprotein particle formation. Type VI patients can include Abetalipoproteinemia (ABL) due to mutations in the microsomal transfer protein, and Homozygous hypobetalipoproteinemia (HHBL) due to structural mutations in apoB. Both disorders lead to severely impaired chylomicron formation and malabsorption of fat-soluble vitamins. These patients should be treated with high dose Vitamin A and E to prevent vision loss and other neurological problems [27]. Individuals with more benign disorders of low apoB, who usually do not develop fat soluble vitamin deficiencies, such as patients with Familial Hypobetalipoproteinemia (FHBL) due to heterozygous mutations in apoB or Familial Combined Hypolipidemia due to mutations in ANGPTL3, can also have low NonHDL-C and TG, but they usually have values outside the range we used to define Type VI [28].

**Fig. 2:**
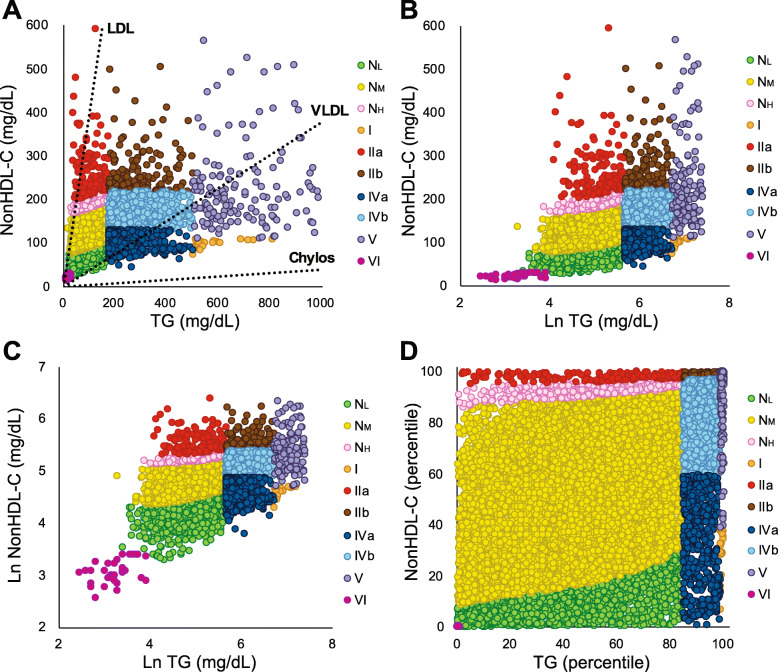
Lipoprotein phenotyping of subjects by new classification system. Samples (*N* = 11,365) from patients seen at the NIH, who were categorized into phenotypes based on NonHDL-C and TG rules (**A**) or as NonHDL-C and natural log TG (**B**) or as natural log NonHDL-C and natural log TG (**C**). NonHDL-C and TG were converted into percentiles and plotted as the percentile NonHDL-C versus percentile TG (**D**). Each point represents an individual sample. Phenotypes are color coded as indicated. Dotted lines in Panel A indicate cholesterol-to-TG ratio of indicated purified lipoproteins (LDL-C = 4, VLDL-C = 0.4, Chylos = 0.04)

To characterize the lipoprotein profile of subjects classified into our new lipoprotein phenotypes, we performed NMR analysis on patients (*N* = 11,365) that underwent routine diagnostic testing at the NIH clinical laboratory. Each patient was first classified into their lipoprotein phenotype by plotting their nonHDL-C values against TG (Fig. 2A). Each point in the plot corresponds to an individual patient and represents the concentration of cholesterol and TG that are contained within the different types of apoB-containing lipoproteins present in a sample. The 3 dotted lines in Fig. 2A correspond to the typical cholesterol-to-TG ratio for purified LDL, VLDL or chylomicrons [2]. As TRL particles undergo TG loss via lipolysis and gain cholesterol from cholesteryl ester transfer protein (CETP)-mediated lipid exchange [29], they transition from the lower right corner of the plot (TG-enriched) to the upper left corner (cholesterol-enriched). Note that even for the Type I phenotype, all points fall well above the dotted line representing pure chylomicrons. Although Type I patients have relatively high levels of chylomicrons compared to other phenotypes, they still have a greater number of LDL and VLDL particles, which causes their position on the graph to shift above the pure chylomicron line. Similarly, most patients with Type IIa, with an increase in cholesterol-enriched LDL particles, mostly fall to the right and below the dotted line representative of pure LDL because they also contain other types of lipoprotein particles, such as VLDL that are more enriched in TG.

To better visualize the Type VI phenotype, we plotted NonHDL-C versus the natural log of TG (Fig. 2B) and also the natural log of NonHDL-C versus the natural log of TG (Fig. 2C). Out of the 34 patient samples identified as having the Type VI phenotype, we were able to confirm that 33 of them came from either 3 different ABL patients, with undetectable apoB (< 10 mg/dL), or from 4 different HHBL patients, who all had an apoB< 13 mg/dL. One patient identified as Type VI, however, had Familial Combined Hypolipidemia, with an apoB of 34 mg/dL. Thus, apoB testing is recommended to confirm the diagnosis of ABL or HHBL in all Type VI patients.

To facilitate the visualization of phenotype frequencies and how they relate to each other metabolically, the concentrations of NonHDL-C and TG were converted into percentiles (Fig. 2D). Most of the plot is occupied by the 3 normolipidemic phenotypes, whereas all the abnormal phenotypes fall into two peripheral zones. The most extreme phenotypes with elevated NonHDL-C or TG or both greater than at least the 97.5th percentile fall along the most extreme top and right edges of the graph. On the top edge where NonHDL-C is the highest are the locations of the Type IIa and IIb phenotypes. Along the right edge of the plot where TG is the highest are the Type I and V phenotypes. The two mixed dyslipoproteinemias, Type IIb and V, fall in the top right corner of the plot where both NonHDL-C and TG are simultaneously elevated. Just inside the most extreme lipoprotein phenotypes are the N_H_, Type IVb and Type IVa phenotypes, which have more intermediate elevations of NonHDL-C and or TG that are between the 85th and 97.5th percentile. Among all of the abnormal phenotypes, Type IV is by far the most common (Fig. 2D), particularly Type IVb (8.3% in NIH database) (Table 1). When we examined a more general NHANES population (Table 1), Type IVb was even more common at 12.5% of the total population. It is also worth noting that Type IIb was slightly more frequent in NHANES than Type IIa (2.1 vs 1.6%). Both Type I and VI are very rare phenotypes; no one with Type I was detected in NHANES and only two with Type VI. Out of the 20 samples in the NIH population that we identified as having Type I, 18 came from 4 different patients diagnosed with lipodystrophy, a known cause of hyperchylomicronemia [30].

**Table 1:** Frequency of phenotypes in NIH and NHANES populations and mean lipid values for NIH population

Phenotypes	NIH (*N* = 11,365)	NHANES (*N* = 13,086)	TC		TG		HDL-C		LDL-C^4^		NonHDL-C		ApoB	
N_L_	1600 (14.1%)^1^	1004 (7.71%)^1^	126(112–139)^2^	G^3^	84 (57–110)	H	53 (40–65)	B	56 (49–64)	G	73 (65–81)	G	63 (55–70)	G
N_M_	7455 (65.6%)	8742 (66.8%)	180 (160–201)	E	91 (66–117)	G	56 (45–67)	A	106 (89–123)	E	124 (106–143)	E	94 (82–106)	E
N_H_	370 (3.3%)	703 (5.4%)	251 (239–262)	C	104 (80–128)	F	58 (49–68)	A	172 (165–178)	C	192 (185–199)	C	137 (129–144)	C
I	20 (0.2%)	0 (0%)	121 (113–128)	FG	577 (535–618)	B	20 (12–29)	F	−15 (−26--4)	I	100 (93–107)	F	80 (68–92)	F
IIa	166 (1.5%)	205 (1.6%)	313 (280–346)	A	108 (76–139)	F	56 (46–66)	AB	235 (206–265)	A	257 (230–283)	A	176 (158–194)	A
IIb	153 (1.3%)	274 (2.1%)	312 (279–345)	A	292 (230–354)	C	47 (38–56)	C	206 (181–231)	B	265 (236–293)	A	181 (162–200)	A
IVa	483 (4.2%)	391 (3%)	141 (123–159)	F	245 (203–286)	E	36 (27–45)	E	56 (39–72)	G	105 (91–119)	F	80 (68–92)	F
IVb	940 (8.3%)	1642 (12.5%)	206 (187–225)	D	260 (208–312)	D	39 (32–46)	D	115 (97–132)	D	167 (150–183)	D	120 (107–133)	D
V	144 (1.3%)	123 (0.9%)	261 (209–314)	B	686 (580–792)	A	32 (24–40)	EF	92 (43–142)	F	229 (182–277)	B	144 (114–173)	B
VI	34 (0.3%)	2 (0%)	50 (43–58)	H	16 (13–19)	I	28 (24–32)	EF	19 (15–23)	H	22 (18–25)	H	29 (25–34)	H

Footnotes ^1^Numbers in parenthesis indicate % total population

^2^Numbers in parenthesis indicate inter-quartile range

^3^Capital letters indicate differences in group means as determined by ANOVA

^4^LDL-C was calculated by Friedewald equations, which can lead to erroneously negative values for high TG samples

**Fig. 3:**
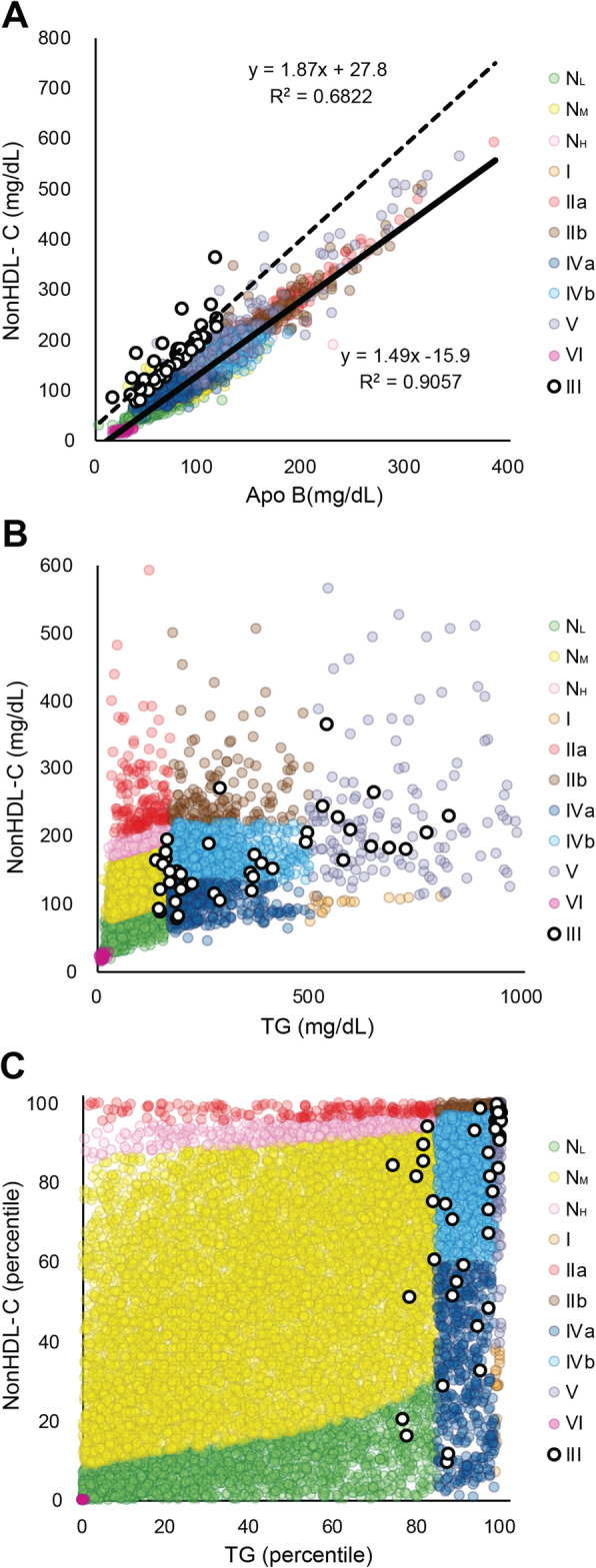
Classification of Type III phenotype by apoB-based decision rules. Regression analysis of NonHDL-C versus apoB for Type III patients (open white circles: dotted line) versus remaining phenotypes (color coded faint circles: solid line) for NIH samples (*N* = 11,365) (**A**). Type III patients identified by the NonHDL-C/apo B rule plus other Sniderman rules for Type III were (open white circles) versus other phenotypes (color coded faint circles) were plotted on graph of NonHDL-C versus TG (**B**). Type III patients identified by NonHDL-C/apo B rule plus other Sniderman rules for Type III (open white circles) were plotted onto percentile plot containing other phenotypes (color coded faint circles) (**C**)

A limitation of our new classification method is its inability to identify Type III dyslipidemia, because of their variable NonHDL-C and TG values that overlap with the other phenotypes. Applying to the NIH database, the NonHDL-C/apoB > 4.91 mmol/g rule [31], which was recently shown to be more specific than the Sniderman rule of TC/apoB> 6.2 mmol/g for identifying Type III [10], we identified a total of 41 Type III patients, approximately 0.4% of our population. Most Type III patients fell into regions of the plot corresponding to Type IVb and V, but they also overlapped with some of the other phenotypes (Fig. 3). We also identified 98 Type III patients by a combination of agarose gel electrophoresis and beta-quantitation from patients (*N* = 5602) at the Mayo Clinic. These 98 Type III patients based on their NonHDL-C and TG values had the following phenotypes by the new classification system: Type IIb 33, Type IVb 23, Type IVa 2, Type V 40. Hence, when a patient presents with clinical features, such as palmar xanthomas, or a history consistent with Type III, additional testing, such as apoB or agarose gel electrophoresis, will be necessary to confirm the diagnosis.

### Validation of new method for lipoprotein phenotype classification

**Fig. 4:**
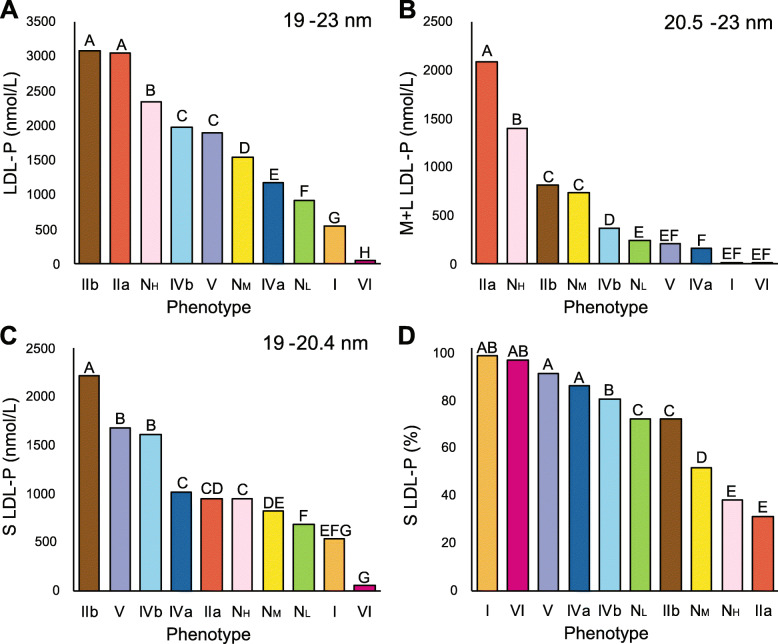
NMR lipoprotein particle parameters for LDL. Samples in NIH database (*N* = 11,365) were analyzed by NMR for (**A**) Total LDL-P, (**B**) Large-medium LDL-P, (**C**) small LDL-P, and (**D**) % small LDL-P. Capital letters indicate differences in group means as determined by ANOVA

**Fig. 5:**
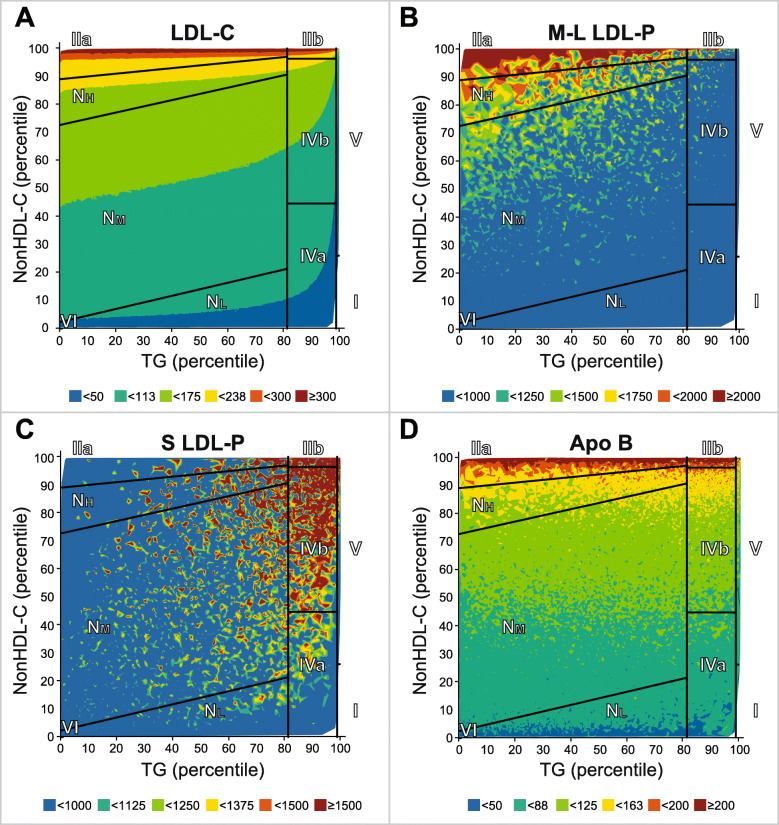
Contour plots for LDL related parameters. Samples in NIH database (*N* = 11,365) were analyzed for the following LDL related parameters and plotted as a contour plot: **(A)** LDL-C, **(B)** Large-medium LDL-P, **(C)** small LDL-P, and **(D)** apoB

The lipid values for our phenotypes are shown in Table 1 and match what would be expected for the classic Fredrickson phenotypes, but we further validated our new classification system by comparing their NMR lipoprotein profile (Fig. 4). Type IIa and IIb had a similarly high level of total LDL-particles (LDL-P), but Type IIa had higher LDL-C concentrations (Table 1). This can also be seen when LDL related parameters were plotted as a contour plot (Fig. 5). Type IIa followed by the N_H_ phenotype had the highest level of large LDL particles, which have a greater cholesterol carrying capacity than small LDL (Figs. 4, 5). In contrast, Type IIb patients had the highest concentration of small LDL particles, followed by Type V and IVb (Figs. 4, 5). In general, all of the high TG phenotypes had a relatively high percent of small LDL particles. This is consistent with the well-known phenomenon of CETP-mediated lipid exchange and subsequent TG lipolysis resulting in the formation of small dense LDL particles [32, 33].

**Fig. 6:**
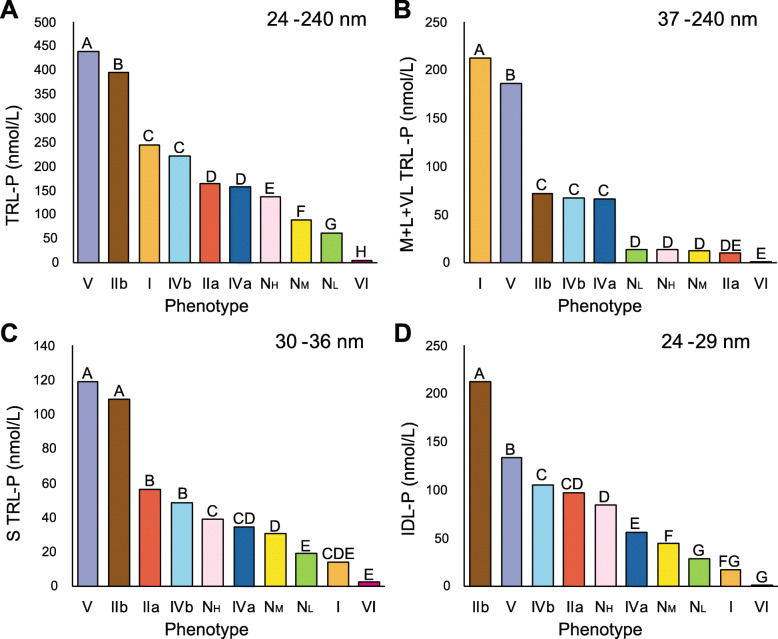
NMR lipoprotein particle parameters for TRL. Samples in NIH database (*N* = 11,365) were analyzed by NMR for **(A)** total TRL-P, **(B)** Very large-large-medium TRL, **(C)** small TRL, and **(D)** IDL. Capital letters indicate differences in group means as determined by ANOVA

Total TRL-particles (TRL-P) were the highest in Type V followed by IIb and I. Type I had the greatest number of large TRL particles (Fig. 6), most likely due to delayed clearance of chylomicrons, which are the most enriched in TG. Many of the TRL particles in Type V were smaller, consistent with having undergone partial lipolysis, as has been previously reported [34]. Small size VLDL particles in the range of 24–29 nM, which are sometimes referred to as Intermediate-Density Lipoproteins (IDL) particles, or VLDL- remnants or simply as remnants were by far the highest in Type IIb (Fig. 6). The mean ApoB level was also the highest in Type IIb (Table 1) but also increased in Type IIa (Fig. 5). Type IVb patients had an intermediate phenotype; they had considerably more TRL-P compared to the N_M_ phenotype, but fewer TRL-P than Type V or IIb. Type IVb patients also had more IDL (Fig. 6) and small LDL particles (Fig. 4) compared to the N_M_ and N_L_ phenotypes. The Type IVa phenotype had fewer overall TRL-particles than Type IVb except for medium and large TRL particles, which were similar in both phenotypes. The Type IVa phenotype also had less total LDL-P than most of the other hyperlipidemic phenotypes except for Type I (Fig. 4A). In terms of HDL, all of the high TG phenotypes (I, IIb, IVa, IVb and V) were low in HDL-C (Table 1) and contained more small HDL particles (Supplemental Fig. 3). In summary, differences in the NMR lipoprotein profile between the different phenotypes are consistent with their known underlying metabolic defects and what has been previously observed by other lipoprotein subfractionation methods.

In Table 2, we further validated the new lipoprotein phenotyping method by comparing it a combination of agarose gel electrophoresis and beta-quantification (gel-lipid method) on patients (*N* = 5602) tested at the Mayo Clinic. For the 3 new normolipidemic phenotypes (N_H_, N_M_, and N_L_), the two lipoprotein classification systems showed over 90% concordance. In the case of the N_H_ phenotype, we scored mild Type IIa by the gel-lipid method as equivalent, because they have nearly identical selection criteria (see methods). There was only one Type I case identified by the new classification method, but it was scored as Type V by the gel-lipid system. All 530 patients identified as Type IIa by the new classification method were classified the same way by the gel-lipid method. In the case of Type IIb by the new classification method, approximately one quarter of the cases (*N* = 121/416) were discordantly classified as Type IIa by the gel-lipid method. Although TG concentrations were significantly lower for these discordant cases compared to concordant Type IIb cases (205 ± 34 mg/dL vs. 296 ± 77; *p* < 0.001), all of the Type IIa discordant cases had TG ≥ 175 mg/dL, causing them to be classified as Type IIb by the new classification method, which is more consistent with how hypertriglyceridemia is currently defined by the 2018-Multisociety Guideline [1]. All of the remaining discordant cases that were classified as Type IV by the gel-lipid method (*N* = 19) had NonHDL-C > 220 mg/dL but also had relatively low levels of LDL-C as measured by beta-quantification, which accounts for the differences in the two classifications. Given that it is now generally recommended by current guidelines to use NonHDL-C rather than LDL-C as an ASCVD biomarker when TG is elevated, these discordant cases are also perhaps best considered as Type IIb, which again is more congruent with the new classification method.

**Table 2:** Comparison of new lipoprotein phenotype classification method with gel-lipid method

New Phenotypes	Gel-Lipid Phenotypes				TG	TG	TG	NonHDL-C	NonHDL-C	NonHDL-C	BQ-LDL-C	BQ-LDL-C	BQ-LDL-C
		N	% Total	Concordance	Mean	Min	Max	Mean	Min	Max	Mean	Min	Max
**N**_**L**_		268		94	90 ± 32	30	174	77 ± 9	49	98	65 ± 8	32	82
	Normal	252	**94**		86 ± 26	30	149	76 ± 9	49	94	65 ± 8	40	82
	Mild Type IV Hyperlipoproteinemia	16	6		168 ± 5	160	174	83 ± 13	56	98	60 ± 12	32	76
**N**_**M**_		1624		94	89 ± 30	26	174	117 ± 20	79	190	104 ± 19	67	169
	Normal	1521	**93.7**		85 ± 27	26	149	114 ± 15	79	152	101 ± 14	67	129
	Mild Type IV Hyperlipoproteinemia	26	1.6		169 ± 5	154	174	127 ± 18	99	159	101 ± 18	67	127
	Mild Type IIb Hyperlipoproteinemia	11	0.7		170 ± 4	162	174	165 ± 9	154	180	141 ± 9	131	157
	Mild Type IIa Hyperlipoproteinemia	66	4.1		116 ± 34	53	173	177 ± 7	163	190	163 ± 2	160	169
**N**_**H**_		702		93	113 ± 32	34	174	194 ± 10	170	222	175 ± 9	154	199
	Mild Type IIa Hyperlipoproteinemia	655	**93.3**		112 ± 31	34	174	193 ± 10	170	222	174 ± 8	160	189
	Type IIa Hyperlipoproteinemia	45	6.4		122 ± 32	62	173	208 ± 7	195	222	193 ± 3	190	199
	Mild Type IIb Hyperlipoproteinemia	2	0.3		173 ± 1	172	174	203 ± 5	199	206	164 ± 13	154	173
**I**		1		0	614	614	614	101	101	101	40	40	40
	Type V Hyperlipoproteinemia	1			614	614	614	101	101	101	40	40	40
**IIa**		530		100	113 ± 33	32	174	246 ± 44	198	562	225 ± 43	176	540
	Type IIa Hyperlipoproteinemia	530	**100**		113 ± 33	32	174	246 ± 44	198	562	225 ± 43	176	540
**IIb**		416		100	282 ± 87	175	494	261 ± 47	220	601	204 ± 49	83	593
	Type IIb Hyperlipoproteinemia	276	**66.3**		296 ± 77	181	492	259 ± 42	220	499	204 ± 41	134	429
	Type IIa Hyperlipoproteinemia	121	**29.1**		205 ± 34	175	389	260 ± 52	220	601	224 ± 55	172	593
	Type IV Hyperlipoproteinemia	19	**4.6**		436 ± 41	356	494	236 ± 10	221	258	143 ± 13	113	163
**IVa**		354		99	227 ± 51	175	484	110 ± 16	39	129	77 ± 17	17	108
	Mild Type IV Hyperlipoproteinemia	353	**99.7**		226 ± 50	175	484	110 ± 16	39	129	77 ± 17	17	108
	Type V Hyperlipoproteinemia	1	0.3		382	382	382	82	82	82	36	36	36
**IVb**		1281		100	260 ± 72	175	499	176 ± 25	130	219	132 ± 27	53	197
	Type IV Hyperlipoproteinemia	629	**49.1**		278 ± 81	175	499	157 ± 20	130	218	110 ± 14	53	148
	Mild Type IIb Hyperlipoproteinemia	558	**43.6**		246 ± 58	175	493	191 ± 16	154	219	150 ± 14	130	190
	Mild Type IIa Hyperlipoproteinemia	91	**7.1**		206 ± 34	175	319	204 ± 9	183	219	172 ± 8	160	189
	Type IIa Hyperlipoproteinemia	3	0.2		179 ± 3	177	183	216 ± 3	213	219	193 ± 4	190	197
**V**		328		98	1791 ± 1560	502	11,350	332 ± 170	115	1128	91 ± 54	15	317
	Type V Hyperlipoproteinemia	218	**66.5**		2424 ± 1622	588	11,350	349 ± 188	115	1040	65 ± 31	16	236
	Type IV Hyperlipoproteinemia	84	**25.6**		719 ± 279	502	1829	244 ± 73	125	531	102 ± 32	30	195
	Type IIb Hyperlipoproteinemia	21	**6.4**		652 ± 171	503	1105	319 ± 66	224	491	206 ± 49	158	317
	Type I Hyperlipoproteinemia	5	1.5		4545 ± 2459	2745	8495	456 ± 397	192	1128	72 ± 62	15	160

Type IVb cases by the new classification were discordantly scored about half the time as either mild Type IIb, mild Type IIa or as Type IIa by the gel-lipid method. All of these discordant cases had higher levels of NonHDL-C than the concordant Type IV cases (Table 2), but nevertheless had NonHDL-C levels below 220 mg/dL and TG ≥ 175 mg/dL, leading them to be categorized as Type IVb by the new classification system. Type IVa cases by the new method were classified as mild Type IV by the gel-lipid method in 99% of cases, which can be considered equivalent based on their similar selection criteria (see methods). Finally, Type V cases were concordant in 59% (218/328) of cases. The discordant cases were mostly classified as Type IV (84/328) based on the lack of abundant chylomicrons near the origin of the gel, which can also be an artifact from loss of these large lipoprotein particles from the loading of the gel during electrophoresis. Some of the discordant Type IIb (21/328) had TG ≥ 500 mg/dL, and therefore, are probably best reconsidered as Type V, according to current guidelines because of their increased risk for pancreatitis. In summary, several of the seemingly discordant cases mostly differed in only their name and not their selection criteria (N_H_ vs. mild IIa, Type IVb vs. mild IV). In other instances, discordance was likely due to a combination of subtle differences in the qualitative interpretation of the lipoprotein patterns on the agarose gels and to differences in the type of criteria used over the years for classifying patients into the different Fredrickson phenotypes (qualitative vs quantitative, percentile vs absolute concentration units, NonHDL-C vs LDL-C, different TG cut-points) [35]. When the remaining discordant cases between the new classification method and the gel-lipid method were reclassified based on their plasma TG level, using currently recommended TG-cutpoints, it leads to greater than 90% concordance for the two classifications for almost all of the phenotypes (Table 2).

### Association of lipoprotein phenotypes with ASCVD

We investigated the association of the different phenotypes by the new classification system with other risk factors in the MESA cohort, except for Type I, which was not present in this population (Table 3). As would be expected, because elevated TG is one of the criteria for metabolic syndrome, we found that metabolic syndrome was strongly associated with all of the high TG phenotypes. Other features seen with metabolic syndrome that can increase ASCVD risk, such as diabetes, increased BMI and elevated systolic blood pressure, were also associated with some of the high TG phenotypes, but these associations were not as strong. Differences in C-Reactive Protein (CRP) levels between the phenotypes were relatively small or insignificant. African American males and females comprised 28% of the total population in MESA, but they were about half as frequent in the high TG phenotypes but were more likely to be in the N_L_ and IIa phenotypes (Table 3). Gender was almost equally distributed among the various phenotypes.

**Table 3:** Association of new lipoprotein phenotypes with ASCVD risk factors in MESA

**Phenotypes**	**Number**	**Mean Age**	***p*** **= 0.0049**	**Mean BMI**	***P*** **< 0.0001**	**Mean SBP**	***P*** **= 0.0011**	**Mean CRP**	***P*** **= 0.0097**		
N_L_	246	63 (54–72)	A	28 (23.9–32.1)	C	124 (109–140)	D	3.8 (1.8–5.8)	BC		
N_M_	4632	62 (53–71)	A	28 (24.7–31.4)	C	126 (112–141)	CD	3.7 (2.0–5.3)	C		
N_H_	424	62 (54–69)	ABC	28.5 (25.5–31.5)	BC	127 (113–140)	BCD	3.3 (1.7–4.9)	C		
IIa	91	62 (53–70)	ABC	27.7 (24.9–30.6)	C	126 (111–141)	ABCD	3.4 (1.6–5.2)	BC		
IIb	177	62 (54–69)	ABC	29.4 (26.5–32.3)	AB	129 (115–143)	ABC	3.7 (2.1–5.3)	BC		
IVa	144	62 (54–70)	AB	30 (27.2–32.9)	A	132 (117–146)	A	5.2 (2.1–8.4)	A		
IVb	1040	61 (53–69)	BC	29.4 (26.3–32.5)	A	129 (115–142)	AB	4.2 (2.4–5.9)	BC		
V	34	58 (53–63)	C	29.1 (25.8–32.4)	ABC	131 (116–147)	ABCD	4 (1.6–6.3)	ABC		
**Level**	**Number**	**%AA**	***P*** **< 0.0001**	**% with diabetes**	***P*** **< 0.0001**	**% on BP meds**	*p* **< 0.0001**	**% with Metabolic** **Syndrome**	***p*** **< 0.0001**	**% of Smokers**	***p*** = **0.0002**
N_L_	246	41.1%	A	31.7%	BC	49.6%	A	31.7%	D	19.9%	AB
N_M_	4632	31.0%	B	23.1%	D	36.4%	D	24.7%	E	13.5%	C
N_H_	424	31.1%	B	23.6%	D	29.0%	E	26.4%	DE	15.8%	BC
IIa	91	39.6%	AB	28.6%	CD	23.1%	E	25.3%	DE	15.4%	BC
IIb	177	11.3%	CD	39.0%	BC	40.1%	BCD	76.3%	BC	16.9%	BC
IVa	144	20.8%	C	40.3%	B	49.3%	AB	84.7%	AB	20.1%	AB
IVb	1040	11.3%	D	36.1%	BC	40.2%	C	76.8%	C	17.1%	B
V	34	14.7%	CD	67.6%	A	41.2%	ABCDE	94.1%	A	32.4%	A
**Level**	**Number**	**% Male**	***p*** **= 0.3353**								
N_L_	246	46.3%	A								
N_M_	4632	46.5%	A								
N_H_	424	46.9%	A								
IIa	91	42.9%	A								
IIb	177	46.9%	A								
IVa	144	48.6%	A								
IVb	1040	50.9%	A								
V	34	52.9%	A								

Footnote: *Capital letters indicate differences in group means as determined by ANOVA. Numbers in parenthesis indicate interquartile ranges

**Fig. 7:**
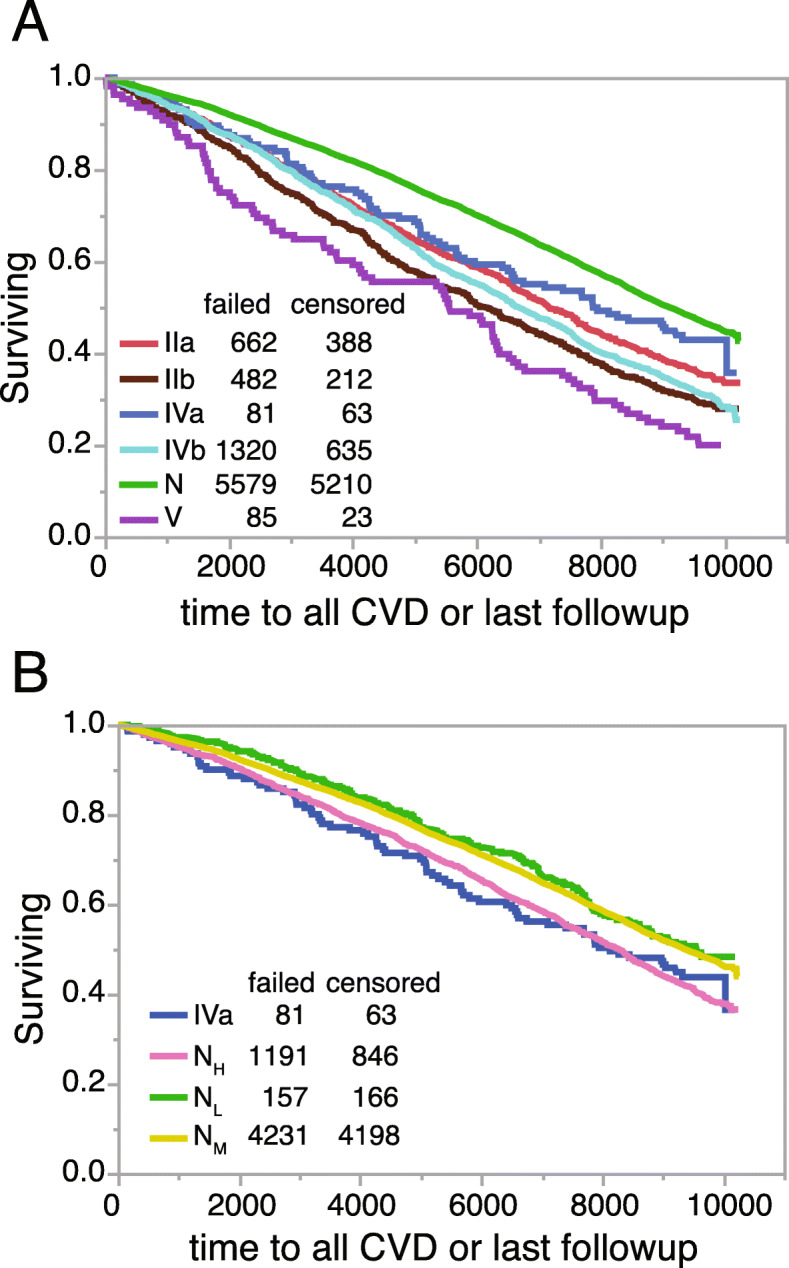
Survival curve analysis by lipoprotein phenotypes. Survival curves in ARIC (*N* = 14,742) for all ASCVD events were calculated for the indicated lipoprotein phenotypes **(A** and **B)**. For ARIC only baseline lipid results from the first study visit were used for analysis and ASCVD was defined as including the following: fatal and non-fatal myocardial infarction, revascularization, stroke and heart failure

Next, we performed ASCVD survival curve analysis for the different phenotypes in ARIC instead of MESA because of its larger size and longer follow-up period (Fig. 7). To simplify the plot, the 3 normolipidemic phenotypes were initially combined and Type I was excluded because of its small sample size (*N* = 2). Type V had the highest rate of ASCVD events, followed by Type IIb and IVb, which were nearly equal, and then Type IIa and IVa and finally the normolipidemic phenotypes (Fig. 7A). In Fig. 7B, we separated the three normolipidemic phenotypes and found as expected that N_H_ showed greater risk than the N_M_ and N_L_, which overlapped. The N_H_ survival curve also overlapped with the curve for the Type IVa phenotype. Finally, we did survival curve analysis for Metabolic Syndrome and found that several of the dyslipidemic phenotypes (Type IIb, IVb and V) had a similar if not a stronger association with ASCVD than Metabolic Syndrome (Supplemental Fig. 4, Supplemental Table 1). This suggests that like Metabolic Syndrome the high-risk lipoprotein phenotypes could potentially be used prognostically as risk enhancer conditions for those patients at intermediate risk as determined by the 10-year risk calculator [1].

## Discussion

Our new lipoprotein phenotyping method (1) enables the classification of all dyslipidemias except for Type III, using standard lipid panel test results, (2) demonstrates an association between some of the lipoprotein phenotypes with ASCVD risk factors, such as diabetes and metabolic syndrome, and (3) revealed for several of the phenotypes a strong association with ASCVD events and hence could be used as risk enhancer conditions. Moreover, new lipoprotein phenotypes could potentially be automated and reported by clinical laboratories on all patients with a standard lipid panel. This may be especially valuable in identifying patients with high-risk mixed dyslipidemias, who are often missed or under-treated [36]. The overall genetic, prognostic, diagnostic and therapeutic implications for the different lipoproteins phenotypes and how it adds value to the standard lipid panel in guiding the clinical management of patients are described below and in Table 4.

**Table 4:** Lipoprotein Phenotypes: Clinical findings and impact on management of patients (ref. cited in table [37–39])

Lipoprotein Phenotypes*	Possible Clinical Findings	Dietary and Pharmacological Treatments	Possible Referrals	Additional Lab Tests to consider	Primary Causes ^	Possible Secondary Causes
***Type I*** ***Chylomicronemia*** ↑↑↑ TG	- Acute Pancreatitis - Eruptive Xanthomas - Lipemia Retinalis - Mental Status Changes	Very low-fat diet, Fibrates, Fish Oil, Niacin	Nutritionist. Consider Lipidologist for refractory cases.	LPL activity assay. Consider apoC-II mutation testing after excluding secondary causes of TG > 885 mg/dL in pediatric populations [40].	**Familial Hyperchylomicronemia** (Autosomal Recessive: LPL, APOC2, GPIHBP1, APOA5, LMF1)	- Type 2 Diabetes - Obesity - Hypothyroidism - Oral Estrogens - Pregnancy - Polycystic Ovarian Syndrome - Alcohol Intake - Chronic Kidney Disease - Non-alcoholic Fatty Liver Disease - Systemic Lupus Erythematosus - Monoclonal Gammopathies - Antiretroviral Therapy - Beta-blockers - Retinoic Acids - Anti-GPIHBP1 antibodies - Lipodystrophy
***Type IIa Hypercholesterolemia*** ↑↑↑ NonHDL-C	- Xanthelasmas - Tuberous Xanthomas - Tendinous Xanthomas - Corneal Arcus	Statins (first line), Ezetimibe, Bile acid resins, PCSK9 inhibitors	Lipidologist, especially for homozygous FH or statin-refractory cases	Consider genetic testing after excluding secondary causes for LDL-C exceeding: a) 190 mg/dL for age < 20 b) 220 mg/for age 20–29 c) 250 mg/dL for age > 30 [54,55].	**Familial Hypercholesterolemia** (Autosomal Dominant: LDLR, APOB, PCSK9) (Autosomal Recessive: LDLRAP1)	- Nephrotic Syndrome - Hypothyroidism - Anabolic Steroids - Cholestatic Liver Disease - Retinoic Acids - Atypical Antipsychotics
***Type IIb*** ***Combined hyperlipidemia*** ↑ TG ↑↑↑ NonHDL-C	No specific signs or symptoms	Statins, Fibrates, Fish Oils, Niacin	Lipidologist for statin-refractory cases	ApoB measurement, Advanced lipid testing (eg: NMR)	Unidentified	- Type 2 Diabetes - Obesity - Nephrotic Syndrome - Cholestatic Liver Disease - Wolman’s disease - Polycystic Ovarian Syndrome - Systemic Lupus Erythematosus - HIV infection - Antiretroviral Therapy - Glucocorticoids - Atypical Antipsychotics - Pregnancy - Retinoic Acids
***Type III Dysbetalipoproteinemia*** ↑↑ TG↑↑ NonHDL-C	- Palmar xanthomas (pathognomonic) - Tuberous xanthomas - Peripheral Vascular Disease	Statins, Fibrates, Fish Oils, Niacin	Lipidologist for statin-refractory cases	ApoB measurement, ApoE isoform assay, Beta-quantitation, Lipoprotein Electrophoresis	**Familial Dysbetalipoproteinemia** (Autosomal Recessive of variable penetrance for APOE-e2 variant, but Autosomal Dominant for rare APOE mutations)	- Type 2 Diabetes - Obesity - Alcohol Intake - Hypothyroidism - Glucocorticoids - Chronic Kidney Disease - Menopause - Monoclonal Gammopathies - Systemic Lupus Erythematosus
***Type IVb Hypertriglyceridemia***↑↑ TG ↑↑ NonHDL-C	- Insulin resistance - Metabolic Syndrome - Obesity	Statins, Fibrates, Fish Oils, Niacin	Generally none.	ApoB measurement, Advanced lipid testing (eg: NMR)	Unidentified	- Type 2 Diabetes - Obesity - Alcohol Intake - Hypothyroidism - Glucocorticoids - Chronic Kidney Disease - Oral Estrogens
***Type V Mixed hyperlipidemia***↑↑↑ TG ↑↑ NonHDL-C	- Acute Pancreatitis - Eruptive Xanthomas - Lipemia Retinalis - Mental Status Changes - Insulin resistance - Metabolic Syndrome - Obesity	Very low-fat diet, Fibrates, Fish Oils, Niacin	Lipidologist and Nutritionist	ApoB measurement, Advanced lipid testing (eg: NMR)	Unidentified	- Type 2 Diabetes - Obesity - Alcohol Intake - Hypothyroidism - Glucocorticoids - Chronic Kidney Disease - Oral Estrogens
***Type VI Hypobetalipoproteinemia***↓↓ TG↓↓↓ NonHDL-C	- Steatorrhea - Hepatic steatosis - Failure to thrive - Spinocerebellar Ataxia - Night Blindness - Retinitis Pigmentosa - Bleeding tendency - Acanthocytes	Low-fat diet, Megadose supplementations of Vitamins A and E +/− Vitamin K	Lipidologist, Nutritionist, Ophthalmologist, Neurologist	ApoB measurement, Plasma Vitamin A and E levels, PT/INR	**Abetalipoproteinemia** (Autosomal Recessive: MTP) **Homozygous Hypobetalipoproteinemia** (Autosomal Recessive: APOB)	- Chronic Liver Disease - Malnutrition - Fat malabsorption syndromes

* *TG* Triglycerides; *HDL-C* High Density Lipoprotein-Cholesterol; *LDL-C* Low Density Lipoprotein-Cholesterol; *FH* Familial Hypercholesterolemia; *HIV* Human Immunodeficiency Virus

^ *LPL* Lipoprotein Lipase; *APOC2* apolipoprotein C2; *GPIHBP1* Glycosylphosphatidylinositol Anchored High Density Lipoprotein Binding Protein 1; *APOA5* apolipoprotein A5; *LMF1* Lipase Maturation Factor 1; *LDLR* Low Density Lipoprotein Receptor; APOB = apolipoprotein B; *PCSK9* Proprotein convertase subtilisin/kexin type 9; *LDLRAP1* Low Density Lipoprotein Receptor Adaptor Protein 1; *APOE* apolipoprotein E; *MTP* Microsomal transfer protein

When the Fredrickson classification was first developed, one early hypothesis was that the different lipoprotein phenotypes were due to distinct monogenic mutations, but this is not the case for most phenotypes (Table 4). Even in the case of Familial Hypercholesterolemia (FH) with the Type IIa phenotype for which there are known causal monogenic mutations in the LDL-receptor gene or related genes, most patients with the Type IIa have polygenic hypercholesterolemia [41]. In addition, the lipoprotein phenotype unlike the genotype is not invariant and can change in response to drug therapy and environmental factors, such as diet and physical activity. Nevertheless, establishment of the lipoprotein phenotype may help focus the management of hyperlipidemia [42]. For example, although secondary causes, such as obesity and diabetes, are in general associated with dyslipidemias, several of the secondary causes listed in Table 4 are more specific for certain phenotypes. As described in Table 4, secondary causes should always be considered when first evaluating a patient. Secondary causes can frequently be uncovered by a careful patient history and physical exam and when addressed can often resolve the dyslipidemia.

In terms of primary causes, the more stringent criteria that we applied for identifying Type IIa and some of the other phenotypes than the Sniderman classification should enable a more targeted identification of patients with monogenic mutations. Once the more common secondary causes are excluded, genetic testing of Type IIa patients to identify specific pathogenic mutations (*LDLR, APOB, LDLRAP1, PCSK9*) can be considered, depending on the LDL-C level (Table 4) and can help inform family cascade screening [23]. Patients with extreme elevations of TG (Type I and V) can also have specific pathogenic mutations (e.g. *LPL, APOC2, GPIHBP1, LMF1, APOA5*) [43]. Type V patients, however, who typically present in adulthood with dyslipidemia, are more likely to have multifactorial chylomicronemia with one or more secondary causes, such as uncontrolled diabetes [44]. Polygenic risk scores for both hypercholesterolemia [41] and hypertriglyceridemia [45] have also been described, but their clinical utility is still being investigated. As we do in Table 4 and as recommend by others [46], the designation “Familial” is perhaps best reserved for those cases in which a primary monogenic cause or genotype has been established by DNA sequencing or biochemical testing. Otherwise, the phenotypes can either be referred to by their traditional Roman numeral designations as we did or alternatively by the more generic descriptive terms shown in Table 4 in order to avoid confusion between genotype and phenotype.

Another value to the lipoprotein phenotyping is related to their prognosis and need for referral to a medical specialist. In the cases of Type I and V, referral to a lipid specialist and nutritionist is highly recommended for preventing pancreatitis [47]. It has been shown that patients with markedly elevated TG often have poor follow-up [48]. Identifying such patients by lipoprotein phenotyping, followed by specific and appropriate treatment can reduce the incidence of acute pancreatitis [49] In case of Type I, referral to a nutritionist is important because adherence to a strict low-fat diet is challenging but often necessary as pharmacotherapy often does not add much benefit when lipoprotein lipase activity is very low or nonexistent [34]. Because of the high incidence of ASCVD in patients with Type IIa, IIb, and III phenotypes [12, 33], and their frequent need for combination pharmacotherapy [50], many of these patients should be seen by a lipid specialist, particularly if they do not show a good response to initial therapy. Type VI patients should not only be referred to a lipid specialist but also to a nutritionist for instructions on a low-fat diet and for high-dose fat soluble vitamin supplementation. They may also need additional referrals to an ophthalmologist and neurologist to monitor and or treat the other clinical manifestations of their disorder.

Lipoprotein phenotyping also has diagnostic testing implications. Because small LDL, IDL, and remnant lipoproteins, which are all considered pro-atherogenic, were found by NMR to be enriched in Type IIb, IV, and V, more in depth advance lipid testing, including the measurement of apoB, may be useful for these phenotypes (Table 4). Numerous studies have shown that when TG is elevated that LDL-C, particularly when calculated, underestimates ASCVD risk and that NonHDL-C, apoB, or LDL-P may be better suited for risk assessment [16, 17, 51, 52]. Although no single pathogenic mutation has been associated with Type IIb [36], apoB may be useful to screen Type IIb families with a strong history of ASCVD, because it has been shown to be a better risk predictor in this population than LDL-C [16]. Importantly, ApoB should also be ordered in all Type VI patients to confirm their diagnosis and whenever Type III is suspected based on clinical findings. In case of the N_H_ phenotype, we observed in NHANES that more than 75% of these patients had an apoB> 130 mg/dL, whereas less than 3% of N_M_ phenotype patients did so. This suggests that ordering apoB as a risk enhancer test is less likely to be informative for the N_M_ phenotype, and its main impact in the N_H_ phenotype may be in identifying those patients that have lower not higher risk because of a lower-than-expected apoB value.

Because the lipid cut-points utilized by our phenotyping method are largely based on current lipid guidelines, lipoprotein phenotyping can be an aid in cardiovascular risk assessment and for improving compliance with guidelines. According to 2018-Multisociety Guidelines [1], patients with an LDL-C ≥ 190 mg/dL, which is equivalent to Type IIa in the new classification system, are considered to have sufficient risk to warrant high-intensity statin treatment without requiring calculation of their 10-year ASCVD risk. Thus, the proposed lipoprotein phenotyping method can be useful by automatically identifying and highlighting these high-risk patients. A similar argument can also be made for aggressive statin treatment of all Type IIb patients. Presently, many Type IIb patients may be under-treated unless their nonHDL-C is also considered because their LDL-C is often less than 190 mg/dL [36], which occurred about half the time in our study population. Consistent with what has been previously described [36], we found in ARIC that Type IIb patients had overall an equal if not greater ASCVD risk than Type IIa [36, 52]. In addition, almost all of Type IIb patients in our study had an apoB> 130 mg/dL (96%), a known ASCVD risk enhancer. Furthermore, they are also at increased risk because of their high TG (≥175 mg/dL), another ASCVD risk enhancer. Based on our survival curve analysis, Type V and IVb patients could also be considered high risk patients and considered as risk enhancer conditions for those at intermediate risk by the 10-year ASCVD risk calculation. One group of patients for which ASCVD risk calculation is currently deemed unnecessary because of their low risk are primary prevention patients with an LDL-C < 70 mg/dL. Based on our classification system, this would include all N_L_ patients. It is important to consider, however, particularly for the normolipidemic phenotypes, that other risk factors besides lipids should be considered in determining ASCVD risk.

The classification of patients into different lipoprotein phenotypes can also help direct the choice of therapy (Table 4). For Type IIa and N_H_, the primary lipid abnormality is elevated LDL-C, so statins should be the drug of first choice, if lifestyle modifications prove insufficient. In regard to TG lowering for pancreatitis prevention, fibrates and high dose omega-3 fatty acid supplementation are useful [53]. Lifestyle modifications should also be recommended [25, 26, 54]. For many patients with Type I and some V patients, a very low-fat diet is often an essential part of therapy [50, 54]. Gene therapy for LPL deficiency was approved in Europe, but it is no longer available [55]. Volanesorsen, an anti-sense oligonucleotide against ApoC-III, can lower TG even in patients with Type I from LPL deficiency [56], and is approved in Europe but not in the US [40]. For Type I patients presenting with acute pancreatitis from *APOC2* gene mutations, infusion of fresh frozen plasma from normal donors can be considered for rapidly lowering TG [57].

In treating more moderate forms of hypertriglyceridemia seen in Type IIb, III, and IV, reduction of dietary sugar and ethanol intake, as well as weight reduction and increased physical activity, are often very effective [54]. If these lifestyle measures do not sufficiently lower ASCVD risk, statin therapy should also be added. Statin monotherapy, however, may be insufficient, especially in cases of persistent elevations of LDL-P, small, dense LDL particles or remnant particles [33]. In these cases, full-dose omega-3 fatty acid supplementation in the form of Icosapent ethyl should be considered as it has been shown in the REDUCE-IT trial to reduce ASCVD risk on top of statins in patients with mild to moderate hypertriglyceridemia [13, 58]. Fibrates or nicotinic acid could also be considered [50], but ASCVD benefit has not yet been demonstrated for these drugs in randomized clinical trials when used with statins [14, 33, 50, 59, 60]. With respect to fibrates as add-on to statins, there is only evidence from *post-hoc* analysis demonstrating that fenofibrates are possibly effective [61, 62]. Although it is clear from genetic studies that many of the causes of hypertriglyceridemia increase the risk for ASCVD [61, 63, 64]; it is not known at this time if lowering TG will translate into reduced ASCVD events [61, 65]. Finally, for Type VI patients with ABL and HHBL, it is critical to treat them with high dose Vitamin A and E supplementation to prevent blindness, neuropathy and other clinical problems [27]. A very low-fat diet can also alleviate many of the severe gastrointestinal symptoms from fat malabsorption that almost all these patients develop.

### Study strengths and limitations

A major strength of the study is that our new method for lipoprotein phenotyping has been validated by two different methods (NMR and agarose gel/beta-quantification), in multiple populations (rare lipoprotein disorder patients and community-dwelling individuals). Another major strength of our phenotyping approach is its cost effectiveness. It only relies on the standard lipid panel, thus enabling its rapid implementation in the clinic, without additional cost related to new testing. Furthermore, lipoprotein phenotyping by our method could potentially be automatically performed by clinical laboratory information systems on all patients tested with a standard lipid panel.

A limitation of our approach is that it cannot be used to identify Type III patients; however, Type III is relatively uncommon, and when clinically suspected, additional testing such as apoB, lipoprotein electrophoresis, or beta-quantification can be used to confirm the diagnosis. Another limitation of our method, which is also shared by other lipoprotein phenotype classification systems, is that it does not address disorders related to low HDL and elevated Lp(a) or to the presence of abnormal lipoproteins like LpX. We also acknowledge that the metabolic disturbances that lead to dyslipoproteinemias occur across a continuum, leading to overlap between the phenotypes at the edges. Our lipid cut-points, therefore, are somewhat arbitrary but are almost all based on widely accepted criteria from guidelines that are already used for the management of patients.

## Conclusion

The lipoprotein phenotype is not a specific diagnosis and is instead best viewed like a clinical syndrome. It encompasses a constellation of shared signs and symptoms that is not only useful in ASCVD risk assessment, but also for identifying other lipid-related diseases, directing lipid-lowering therapy, guiding further diagnostic testing and in referring patients to medical specialists. Given that almost all ASCVD drug trials and other related clinical studies will have measured a standard lipid panel, our new classification system can be easily retrospectively applied to many previously performed studies for identifying the association of dyslipidemic phenotypes with various predisposing conditions or for their response to novel drugs or other types of interventions.

## Supplementary Information

**Additional file 1: Supplemental Figure S1.** Flowchart for criteria for new lipoprotein phenotype classification. Using the indicated TG and NonHDL-C decision rules, all Fredrickson phenotypes can be identified except Type III by new classification system.

**Additional file 2: Supplemental Figure S2.** Comparison of apoB versus NonHDL-C. Linear least squares regression analysis of apoB versus NonHDL-C in NHANES (*N*=13,086). Dotted lines indicate equivalent cut-points for both variables.

**Additional file 3: Supplemental Figure S3.** NMR lipoprotein particle parameters for HDL. Samples in NIH database (*N*=11,365) were analyzed by NMR for (A) total HDL-P, (B) Large-medium HDL, (C) small HDL, and (D) % small HDL-P. Capital letters indicate differences in group means as determined by ANOVA.

**Additional file 4: Supplemental Figure S4.** Survival curve analysis by metabolic syndrome status. Survival curves in ARIC (*N*=14742) for all ASCVD events were calculated for the presence of absence of metabolic syndrome. Only baseline lipid results from the first study visit were used for analysis and ASCVD was defined as including the following: fatal and non-fatal myocardial infarction, revascularization, stroke and heart failure.

**Additional file 5: Supplemental Table S1.** Mean survival time for lipoprotein phenotypes and metabolic syndrome.

## Data Availability

De-identified clinical laboratory test results from the National Health and Nutrition Examination Survey (NHANES, 2005–2016) were used for external validation and downloaded from the following website: https://wwwn.cdc.gov/nchs/nhanes/. De-identified datasets for external validation were also obtained from the Multi Ethnic Study of Atherosclerosis (MESA) and the Atherosclerosis Risk in Communities (ARIC), using BioLINCC (https://biolincc.nhlbi.nih.gov/home/). Software developed for lipoprotein phenotyping analysis by the newly developed method can be freely downloaded at the following website: https://figshare.com/articles/software/Sampson_Phenotype_Calculator/16617490

## Abbreviations

ASCVD: atherosclerotic cardiovascular disease
NonHDL-C: Non-High-Density Lipoprotein-Cholesterol
TG: Triglycerides
TC: Total cholesterol
LDL: Low-Density Lipoproteins
VLDL: Very-Low Density Lipoproteins
apoB: Apolipoprotein B
ARIC: Atherosclerosis Risk in Communities
NLA: National Lipid Association
NHANES: National Health and Nutrition Examination Survey
MESA: Multi Ethnic Study of Atherosclerosis
LDL-C_ßQ_: LDL-C as determined by beta-quantification
N_H_: Normolipidemic High risk
N_M_: Normolipidemic Moderate risk
N_L_: Normolipidemic Low risk
ABL: Abetalipoproteinemia
HHBL: Homozygous hypobetalipoproteinemia
FHBL: Familial Hypobetalipoproteinemia
FH: Familial Hypercholesterolemia
CETP: cholesteryl ester transfer protein
LDL-P: LDL-particles
IDL: Intermediate-Density Lipoproteins
TRL: Triglyceride-rich lipoprotein
CRP: C-Reactive Protein
