# Supplementary figures and images for: A new phenotypic classification system for dyslipidemias based on the standard lipid panel
Source: Lipids Health Dis. 2021 Nov 27;20:170. doi: 10.1186/s12944-021-01585-8 (PMC8627634; doi:10.1186/s12944-021-01585-8)

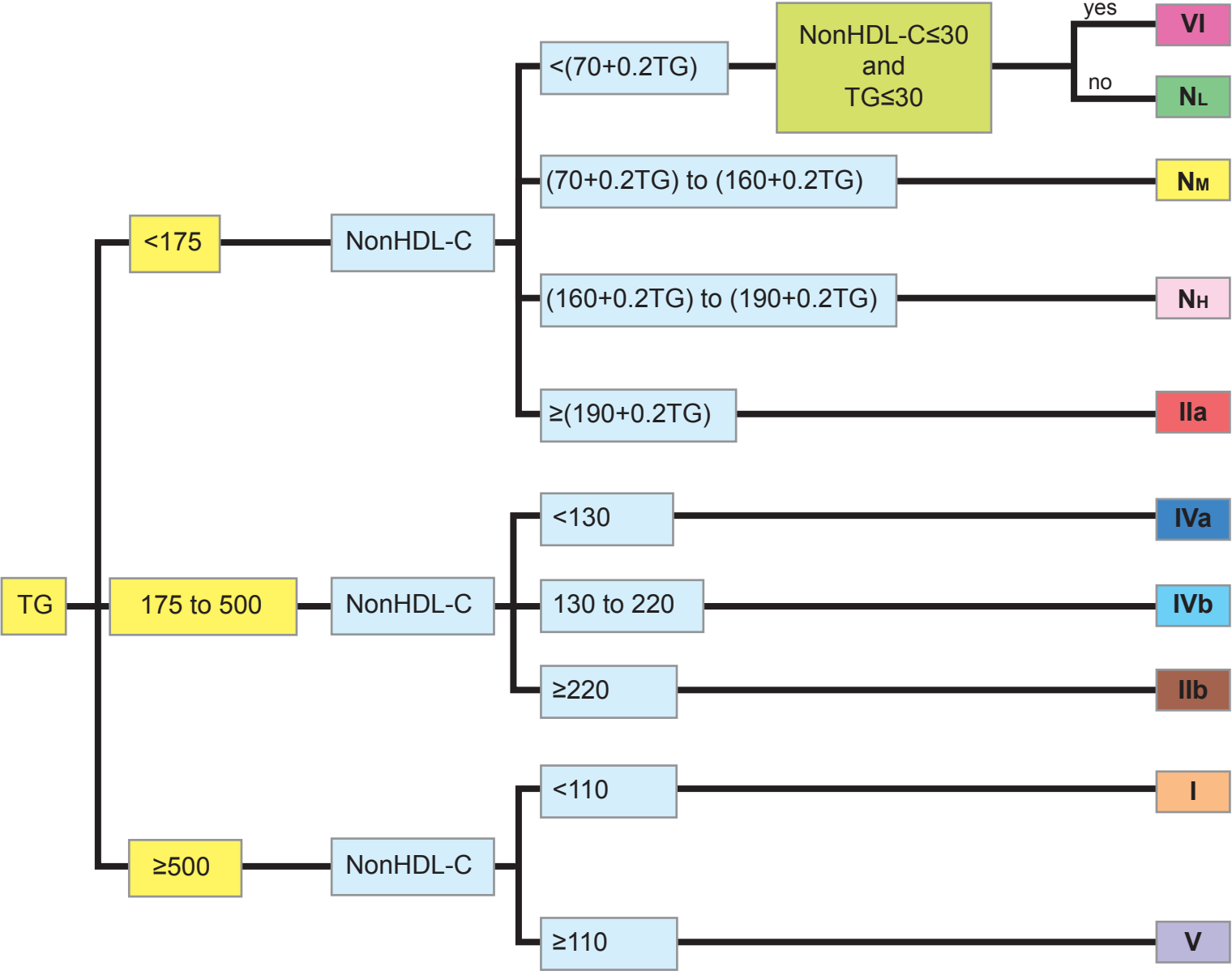

Supplement: Supplementary file 1 — Additional file 1: Supplemental Figure S1. Flowchart for criteria for new lipoprotein phenotype classification. Using the indicated TG and NonHDL-C decision rules, all Fredrickson phenotypes can be identified except Type III by new classification system. [file 12944_2021_1585_MOESM1_ESM.pdf]

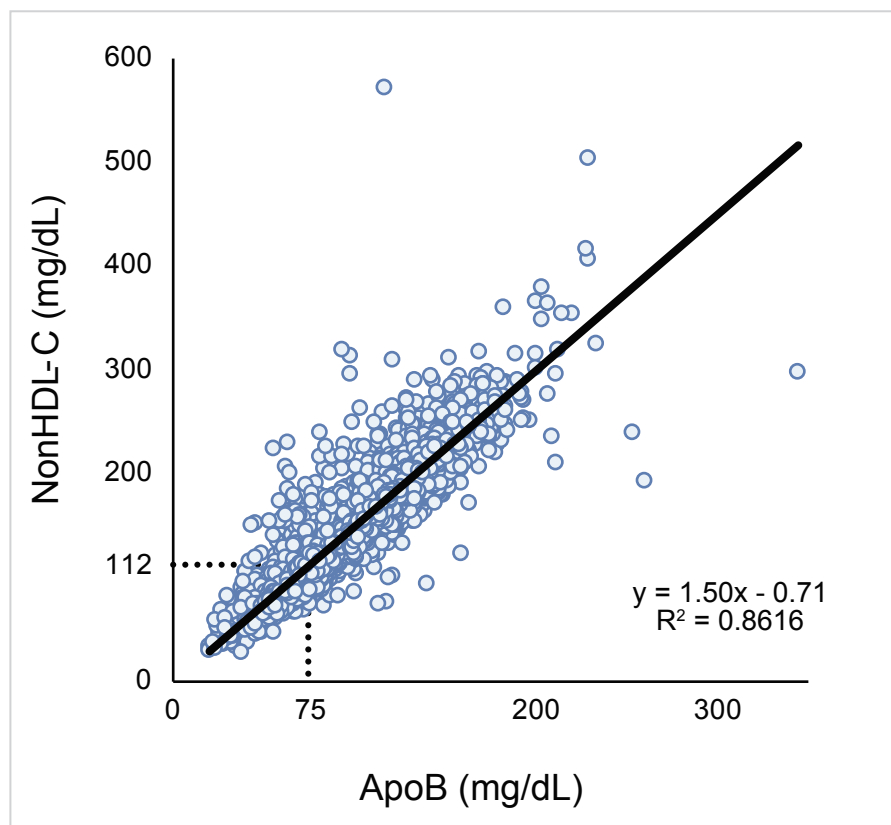

Supplement: Supplementary file 2 — Additional file 2: Supplemental Figure S2. Comparison of apoB versus NonHDL-C. Linear least squares regression analysis of apoB versus NonHDL-C in NHANES (N=13,086). Dotted lines indicate equivalent cut-points for both variables. [file 12944_2021_1585_MOESM2_ESM.pdf]

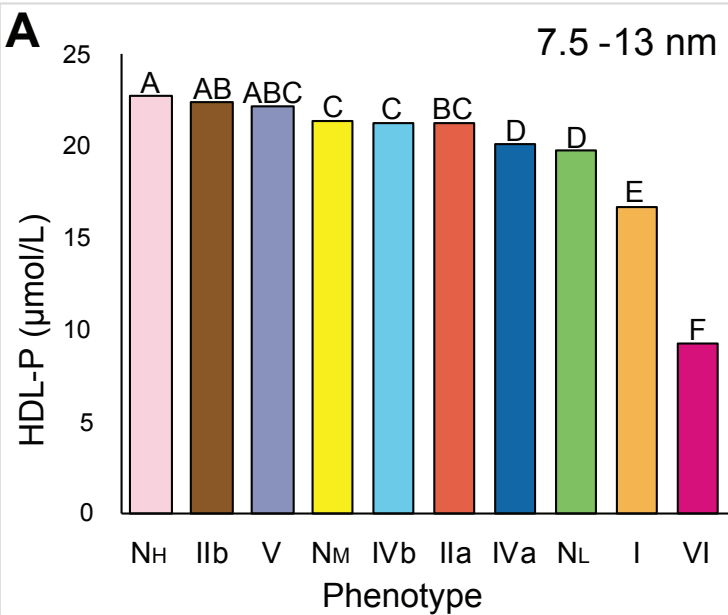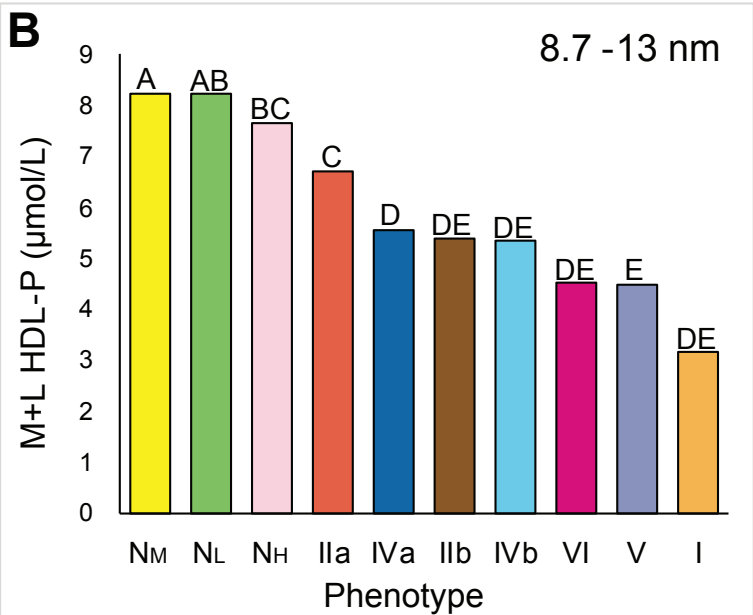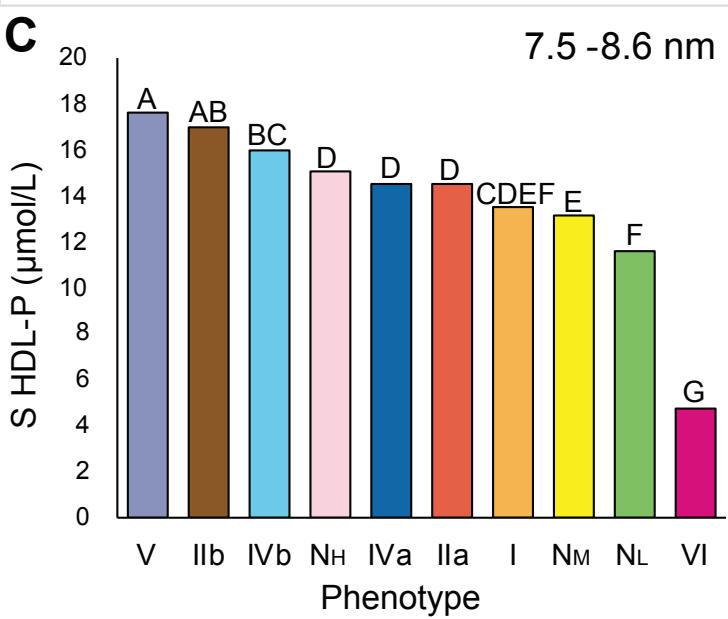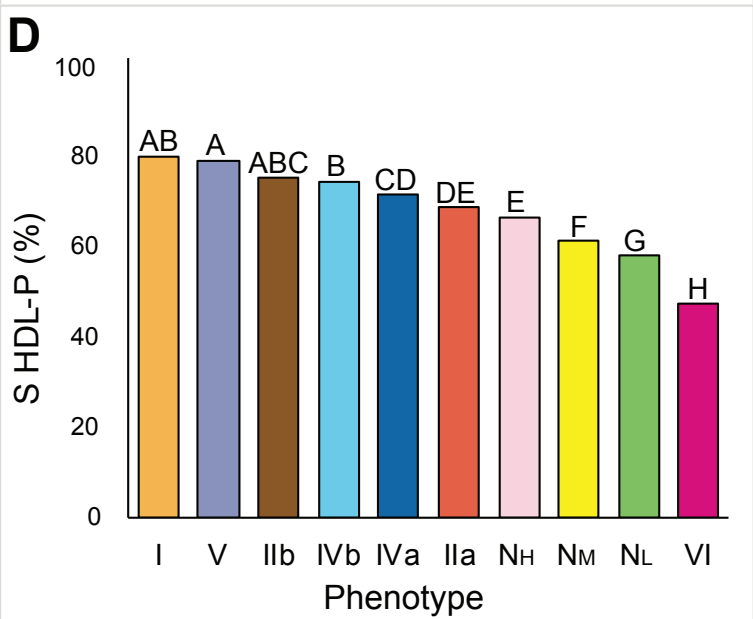

Supplement: Supplementary file 3 — Additional file 3: Supplemental Figure S3. NMR lipoprotein particle parameters for HDL. Samples in NIH database (N=11,365) were analyzed by NMR for (A) total HDL-P, (B) Large-medium HDL, (C) small HDL, and (D) % small HDL-P. Capital letters indicate differences in group means as determined by ANOVA. [file 12944_2021_1585_MOESM3_ESM.pdf]

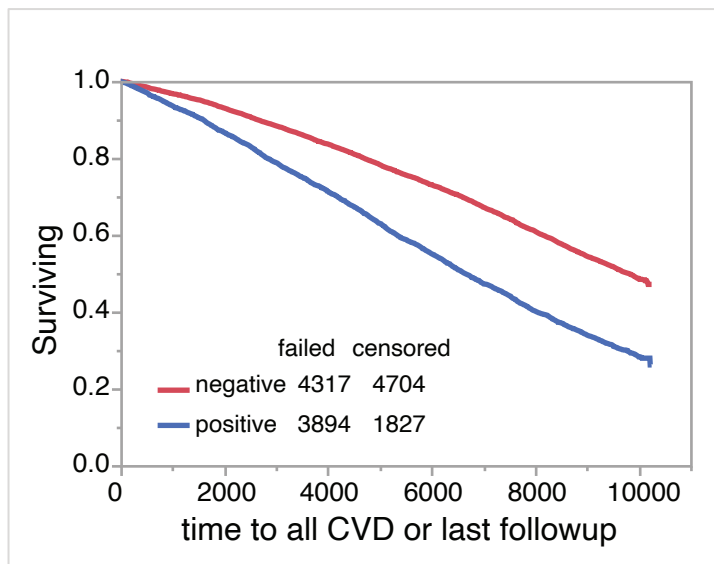

Supplement: Supplementary file 4 — Additional file 4: Supplemental Figure S4. Survival curve analysis by metabolic syndrome status. Survival curves in ARIC (N=14742) for all ASCVD events were calculated for the presence of absence of metabolic syndrome. Only baseline lipid results from the first study visit were used for analysis and ASCVD was defined as including the following: fatal and non-fatal myocardial infarction, revascularization, stroke and heart failure. [file 12944_2021_1585_MOESM4_ESM.pdf]
