# Supplementary material for: A new phenotypic classification system for dyslipidemias based on the standard lipid panel
Source: Lipids Health Dis. 2021 Nov 27;20:170. doi: 10.1186/s12944-021-01585-8 (PMC8627634; doi:10.1186/s12944-021-01585-8)
Supplement: Supplementary file 5 — Additional file 5: Supplemental Table S1. Mean survival time for lipoprotein phenotypes and metabolic syndrome. [file 12944_2021_1585_MOESM5_ESM.docx]

**Supplemental Table 1.** **Mean time to ASCVD event in ARIC for lipoprotein phenotypes and metabolic syndrome status.**

| Phenotype | Mean (days) | Std Err (days) |
| --- | --- | --- |
| N_L_ | 7517 | 155 |
| N_M_ | 7665 | 34 |
| N_H_ | 7132 | 72 |
| IVa | 6985 | 283 |
| IIa | 6595 | 102 |
| IVb | 6432 | 75 |
| IIb | 6022 | 127 |
| V | 5247 | 531 |
| no MetSyn | 7796 | 32 |
| yes MetSyn | 6412 | 44 |
